# Evolution of the Role of RA and FGF Signals in the Control of Somitogenesis in Chordates

**DOI:** 10.1371/journal.pone.0136587

**Published:** 2015-09-15

**Authors:** Stéphanie Bertrand, Daniel Aldea, Silvan Oulion, Lucie Subirana, Angel R. de Lera, Ildiko Somorjai, Hector Escriva

**Affiliations:** 1 UPMC Univ Paris 06, UMR 7232, BIOM, Observatoire Océanologique de Banyuls sur Mer, F-66650, Banyuls/Mer, France; 2 Departamento de Química Orgánica, Facultade de Química, CINBIO, Universidade de Vigo, and Instituto de Investigación Biomédica de Vigo (IBIV), Vigo, Spain; Heart Science Centre, Imperial College London, UNITED KINGDOM

## Abstract

During vertebrate development, the paraxial mesoderm becomes segmented, forming somites that will give rise to dermis, axial skeleton and skeletal muscles. Although recently challenged, the "clock and wavefront" model for somitogenesis explains how interactions between several cell-cell communication pathways, including the FGF, RA, Wnt and Notch signals, control the formation of these bilateral symmetric blocks. In the cephalochordate amphioxus, which belongs to the chordate phylum together with tunicates and vertebrates, the dorsal paraxial mesendoderm also periodically forms somites, although this process is asymmetric and extends along the whole body. It has been previously shown that the formation of the most anterior somites in amphioxus is dependent upon FGF signalling. However, the signals controlling somitogenesis during posterior elongation in amphioxus are still unknown. Here we show that, contrary to vertebrates, RA and FGF signals act independently during posterior elongation and that they are not mandatory for posterior somites to form. Moreover, we show that RA is not able to buffer the left/right asymmetry machinery that is controlled through the asymmetric expression of Nodal pathway actors. Our results give new insights into the evolution of the somitogenesis process in chordates. They suggest that RA and FGF pathways have acquired specific functions in the control of somitogenesis in vertebrates. We propose that the "clock and wavefront" system was selected specifically in vertebrates in parallel to the development of more complex somite-derived structures but that it was not required for somitogenesis in the ancestor of chordates.

## Introduction

Segmentation along the antero-posterior body axis is a morphological feature found in several metazoan lineages. In vertebrates, segmentation is conspicuous in the paraxial mesoderm, which forms transient bilateral symmetric blocks during the somitogenesis process [1]. The somites, which will give rise to the axial skeleton, the skeletal muscles of the trunk and part of the dermis, form in an antero-posterior succession through segmental epithelialisation of the mesenchymal presomitic mesoderm (PSM). During elongation of the vertebrate embryo, a pool of proliferating cells that are continuously added to the caudal zone is maintained in the most posterior part, the tailbud [2]. Although the precise structure of vertebrate tailbuds varies from one species to another, vertebrates share a similar anatomy and the global mechanisms controlling posterior elongation and somitogenesis seem to be conserved. An elegant paradigm was first proposed by Cooke and Zeeman in 1976 to explain the regular formation of segments during somitogenesis termed the "clock and wavefront" model [3]. Molecular evidence for this hypothesis came more than twenty years later and our understanding of how somitogenesis is controlled in vertebrates has been highly improved since then [4, 5]. Our current understanding of the "clock and wavefront" model relies on the specific interactions of several signalling pathways, including the retinoic acid (RA), the Fibroblast Growth Factor (FGF), the Wnt (Wingless/INT-1) and the Notch pathways. These interactions permit the synchronized activation of segmentation genes in the PSM in response to the "segmentation clock" [5]. This clock is defined by periodic waves of expression of genes of the FGF, Wnt, and Notch signalling pathways that are travelling along the PSM [6]. The position of the "wavefront" or "determination front" is defined by the posterior FGF/Wnt pathway which is antagonized by the RA pathway in the rostral region of the PSM [7]. As a consequence of the interaction between the clock and the wavefront, the cells of the PSM that pass the determination border during one oscillation of the clock define a pre-patterned somite [5].

Vertebrates, together with their sister group the tunicates and cephalochordates (i.e. amphioxus), form the chordate superphylum [8, 9]. They share morphological features considered synapomorphies of this clade. Particularly they show, at least transiently during embryonic development, a notochord localized ventral to a dorsal hollow nerve tube. Chordates are also characterized by segmented muscles present on both sides of the main body axis. In tunicates, these muscles are only found in the tail of the tadpole are not formed through the antero-posterior successive segmentation of an unsegmented paraxial mesoderm, but develop directly from muscle cells that are produced early during development and get subsequently rearranged on both sides of the tail midline [10]. In the cephalochordate amphioxus, segmented muscles are derived from somites that form in an anterior-to-posterior sequence, from the most rostral part of the embryo to the most caudal part. However, in contrast to vertebrates, the segmented muscles show a clear asymmetry with the left muscle fibres more anterior than the right ones. The most anterior early-arising somites appear as bilateral pairs by means of enterocoelic evagination of the paraxial dorsal wall of the archenteron, whereas the posterior somites form from the tailbud by schizocoely alternatively on the left and right sides of the embryo [11]. In spite of the morphological differences between amphioxus and vertebrate somitogenesis processes, developing amphioxus somites express homologs of many genes involved at each step of vertebrate paraxial mesoderm segmentation [12]. The only functional evidence of how somitogenesis is controlled in amphioxus came from the analysis of the role of the FGF signalling pathway during embryogenesis [13]. We showed that the formation of the most anterior somites is under the control of FGF whereas posterior somites continue to form in spite of FGFR inhibition [13]. This result suggests that important differences exist in the control of somitogenesis between vertebrates and cephalochordates. However, we still do not know how the formation of somites is governed during posterior elongation in amphioxus and what mechanisms underly the difference in the control of the formation of the most anterior and posterior somites.

In this work, we address these two questions. We show that although *Hox* genes control antero-posterior patterning in amphioxus, and their most anterior limit of expression coincides with the posterior limit of the FGF-sensitive somites [14, 15], they do not define a functional boundary between FGF-sensitive and FGF-insensitive somites. We also reveal that RA is not implicated in amphioxus somitogenesis, either directly or through interaction with the FGF signalling pathway. Moreover, we show that RA is not able to buffer the left/right asymmetry machinery. These data allow us to propose a model for the evolution of the somitogenesis process in which specific vertebrate acquisitions such as the opposition between the FGF and RA pathways or the implication of RA in the control of symmetry played a major role in the evolution of their present morphology.

## Materials and Methods

### Animals, embryo collection and drug treatments

Ripe adults from the Mediterranean amphioxus species (*Branchiostoma lanceolatum*) were collected at the Racou beach near Argelès-sur-Mer, France, (latitude 42° 32’ 53” N and longitude 3° 03’ 27” E) with a specific permission delivered by the Prefect of Region Provence Alpes Côte d’Azur. *Branchiostoma lanceolatum* is not a protected species. Gametes were collected by heat stimulation as previously described [16, 17]. Prior to pharmacological treatments, and before hatching, embryos were transferred to new Petri dishes with a known final volume of sea water. SU5402 (Calbiochem 572630), BMS009 and RA (Sigma R2625) were dissolved in dimethylsulfoxide (DMSO) at 10^−2^ M and added to cultures of embryos at a final concentration of 50x10^-6^ M, 5x10^-6^ M and 10^−6^ M, respectively. The inhibitors of the type I activin receptor-like kinase (ALK) receptors SB505124 (Sigma S4696) and SB431552 (Sigma S4317) were dissolved in DMSO at a final concentration of 10^−2^ M and added to the cultures of embryos at final concentrations of 5x10^-6^ M and 10x10^-6^ M, respectively. Control embryos for all these experiments were raised with 0,5% DMSO in filtered sea water. Omeprazole (Sigma O104) was dissolved in DMSO at 10^−2^ M and embryos were treated at final concentrations varying from 50 x10^-6^ M to 200 x10^-6^ M. Control embryos were raised simultaneously with equivalent concentrations of DMSO. Embryos were staged according to Hirakow and Kajita [18, 19] although at L3 there is only one open pharyngeal slit in *B*. *lanceolatum*.

### *In situ* hybridization and immunostaining

Fixation and whole-mount *in situ* hybridizations were performed as described in [20], but the proteinase K digestion was omitted and we used BM Purple (Roche) for the chromogenic reaction step. The accession numbers of the sequences used for probe synthesis are as in [13, 21, 22] and as follows: *Hu/Elav* (ADU32858), *Sprouty* (ADU32856). Statistical analyses (t-test and one-way ANOVA were undertaken using Excel.

F-Actin staining was performed as described in [13]. Immunostaining was undertaken as described in [23] using primary antibodies against acetylated tubulin (Sigma T6793, 1:500). After three ten-minute washes in NaPBS, embryos were transferred to glycerol with 2,5% DABCO for photographs.

### Statistical analysis

Morphometric data statistical analysis was performed using R. First, a Shapiro-Wilk test was performed to test the normal distribution of the data for each considered group. We then undertook a one-way ANOVA analysis to test if the means of the measurement variables in the groups are significantly different. The resulting p-values are indicated on each figure.

When we obtained p-values < 0.05, we undertook two samples Student t-tests. We adjusted the p-value by using the Bonferonni correction for multiple comparisons.

## Results

### RA/Hox1 and FGF pathways act independently during the formation of the anterior somites

**Fig 1:**
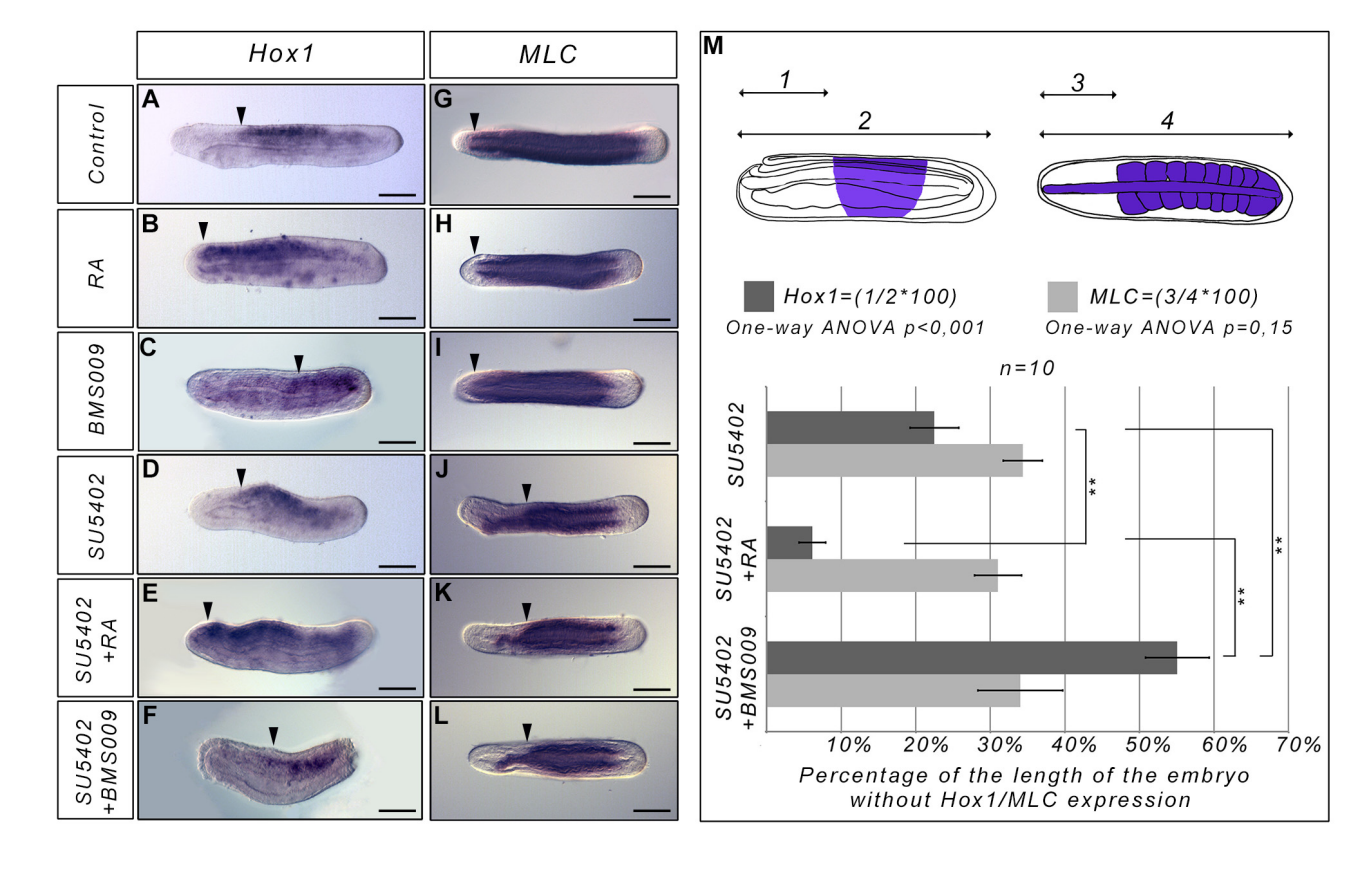
The anterior limit of *Hox1* expression does not define a functional boundary between anterior FGF-sensitive and posterior FGF-insensitive somites. Expression of *Hox1* (lateral views, anterior to the left) and *MLC* (dorsal views, anterior to the left) at the L0 stage in control embryos (A, G), in RA treated embryos (B, H), in BMS009 treated embryos (C, I), in SU5402 treated embryos (D, J), in SU5402 and RA treated embryos (E, K) and in SU5402 and BMS009 treated embryos (F, L). All the treatments were performed at the blastula stage. The arrowheads indicate the anterior limit of Hox1 expression (A-F) or the anterior limit of the embryonic region with formed somites (G-L). (M) Graph presenting the percentage of the length of the embryo without *Hox1* expression (dark grey) or without somites in the anterior region (light grey). The schematic embryos show how this percentage was calculated. (1) corresponds to the length of the anterior region without Hox1 expression, (2) corresponds to the total length of the embryo. (3) corresponds to the length of the anterior region without somite MLC expression. (4) corresponds to the total length of the embryo. Dark blue regions in the schematic embryos correspond to the territories expressing *Hox1* (lateral view, anterior to the left) or *MLC* (dorsal view, anterior to the left) in SU5402 treated embryos. A one-way ANOVA analysis was undertaken and the result indicates that the means of the region without somites is not significantly different between the three treatments (SU5402, SU5402+RA and SU5402+BMS009) whereas the means of the region without *Hox1* expression is significantly different between the three treatment conditions (SU5402, SU5402+RA and SU5402+BMS009). **P<0.003 (corrected p-value); Two samples Student t-test, n = 10 embryos. Error bars indicate s.e.m. Scale bars = 50μm.

We previously showed that inhibiting the FGF signalling pathway at the blastula stage induces a specific loss of the most anterior somites in amphioxus embryos [13]. Strikingly, the anterior paraxial mesoderm region in which somites do not form corresponds to the region where *Hox* genes are not expressed. Thus, we wondered if the necessity for FGF signalling is linked to the anterior limit of *Hox1* expression [22, 24]. To answer this question we treated embryos with RA or BMS009 (an antagonist of the retinoic acid receptor from amphioxus [25]) at the blastula stage and fixed them when the pharynx starts to enlarge (stage L1, 40 h.p.f. at 19°C). As expected, these treatments allowed us to move the anterior border of *Hox1* expression forward or backward, respectively (Fig 1A–1C), whereas the formation of anterior somites is not affected (Fig 1G–1I). We also inhibited the FGF signalling pathway with SU5402 (an inhibitor of FGF receptor [26]) and undertook double treatments with RA and SU5402 or with BMS009 and SU5402. When embryos are treated with both RA and SU5402, the anterior limit of *Hox1* expression is shifted anteriorly compared to embryos only treated with SU5402 (Fig 1D, 1E and 1M). However, the region without somites is comparable to what is observed in embryos in which only FGF signalling is inhibited as highlighted by *Myosin Light Chain-alkali* (*MLC*) expression (Fig 1J, 1K and 1M). Indeed, in the anterior region, we still observe *MLC* expression but it is restricted to the notochord which in SU5402-treated embryos is not straight as in wild-type animals. Moreover, in embryos treated with BMS009 and SU5402, we observe that the anterior limit of *Hox1* expression is shifted posteriorly (Fig 1C, 1F and 1M) but the region without somites is still comparable to what we observe in SU5402 treated embryos (Fig 1J, 1L and 1M). Altogether our data suggest that the anterior border of *Hox1* expression does not define the limit between somites that depend upon FGF signalling for their formation and somites whose formation is not controlled by FGF. Moreover, these data show that there is no cross-talk between RA and FGF signalling for the formation of the most anterior somites in amphioxus.

### FGF and RA signalling pathways act independently during posterior elongation

**Fig 2:**
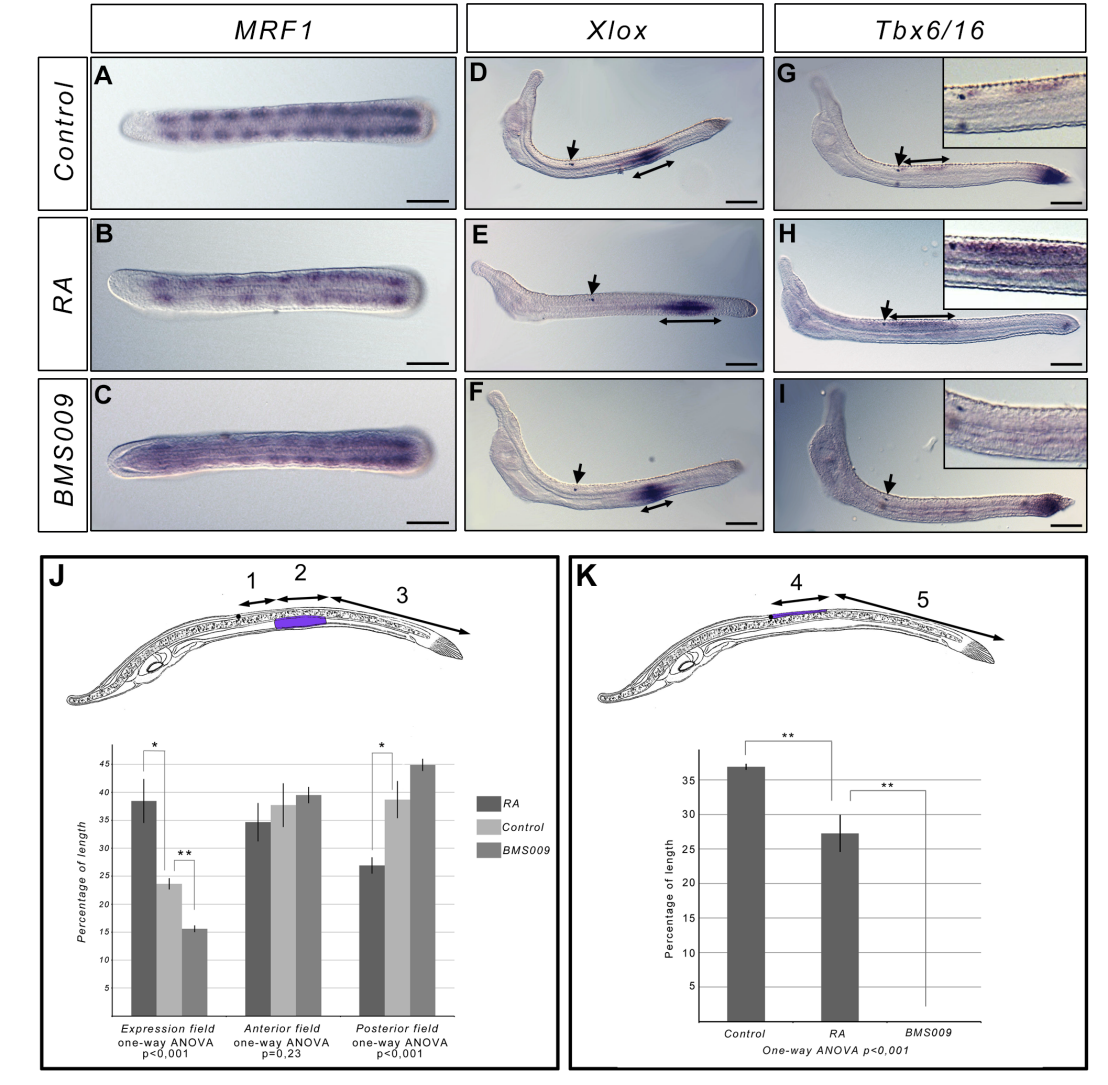
Interfering with RA signalling during posterior elongation does not affect somitogenesis. Expression of *MRF1* in L0 stage control embryos (A), and in embryos treated at the N3 stage with RA (B) or BMS009 (C) (dorsal views, anterior to the left). The expression shows that the number of formed somites is identical in treated and control embryos. Expression of *Xlox* and *Tbx6/16* in L3 stage control embryos (D, G), and in embryos treated at the N3 stage with RA (E, H) or BMS009 (F, I) (side views, anterior to the left). The arrows indicate the position of the pigment spot. The double arrow lines indicate the size of the domain expressing *Xlox* or *Tbx6/16*. Enlargement of the photograph at the level of the pigment spot is presented for *Tbx6/16 in situ* hybridization on the top left of the panels. Scale bars = 50μm. Morphometric analysis of the expression domains of *Xlox* (J) and *Tbx6/16* (K). Schematic larva with the domain of expression highlighted in blue-violet are presented (side view, anetrior to the left). (1) corresponds to the length of the embryo, posterior to the pigment spot, without *Xlox* expression. (2) corresponds to the length of the embryo with *Xlox* expression. (3) corresponds to the length of the posterior field of the embryo without *Xlox* expression. The percentage of the length of the field with *Xlox* expression was calculated as 2/(1+2+3)*100, the percentage of length of the anterior field without expression as 1/(1+2+3)*100 and the percentage of length of the posterior field without expression as 3/(1+2+3)*100 (J). One-way ANOVA analysis indicates that the the means of the percentage of length of the field with *Xlox* expression between the three conditions (control embryos, RA-treated embryos and BMS009-treated embryos) are significantly different as well as the means of the percentage of length of the posterior field without expression of *Xlox* (J). (4) corresponds to the length of the field posterior to the pigment spot showing *Tbx6/16* expression. (5) correspond to the length of the posterior field of the embryo without Tbx6/16 expression. The percentage of length with *Tbx6/16* expression was calculated as 4/(4+5)*100 (K). One-way ANOVA analysis indicates that the the means of the percentage of the length with *Tbx6/16* expression are significantly different between the three conditions (control embryos, RA-treated embryos and BMS009-treated embryos). **P<0.005 (corrected p-value); *P<0,025 (corrected p-value); t-test, n = 3 embryos. Error bars indicate s.e.m.

To test whether the RA signalling pathway is involved in the posterior elongation process, we interfered with this signal at the late neurula stage N3 (27 h.p.f. at 19°C), when 8 to 9 somite pairs are formed. We treated embryos with RA or BMS009. Embryos were fixed at stage L0 and at the larval stage L3 (60 h.p.f. at 19°C) and we analyzed the expression of several marker genes to assess the phenotype of the embryos. The activation of RA signalling induces a reduction of the pharynx size, whereas BMS009 treatment leads to an enlargement of the pharyngeal region as observed when embryos are treated at earlier stages. The formation of new somites is normal with embryos at stage L0 having 11 to 12 somites expressing *MyoD related factor 1 (MRF1*) similarly to control animals (Fig 2A–2C). However, *Xlox* (Fig 2D–2F) and *Tbx6/VegT* (Fig 2G–2I) gene expression show that the antero-posterior patterning is affected in an opposite way by the two treatments in both the endoderm and the neural tube. Indeed, we observe that the expression fields of *Xlox* and *Tbx6/16* are pushed posteriorly in RA treated embryos whereas the posterior domain of expression is pushed anteriorly in BMS009 treated embryos, showing that even at late developmental stages RA signalling is still controlling AP patterning.

**Fig 3:**
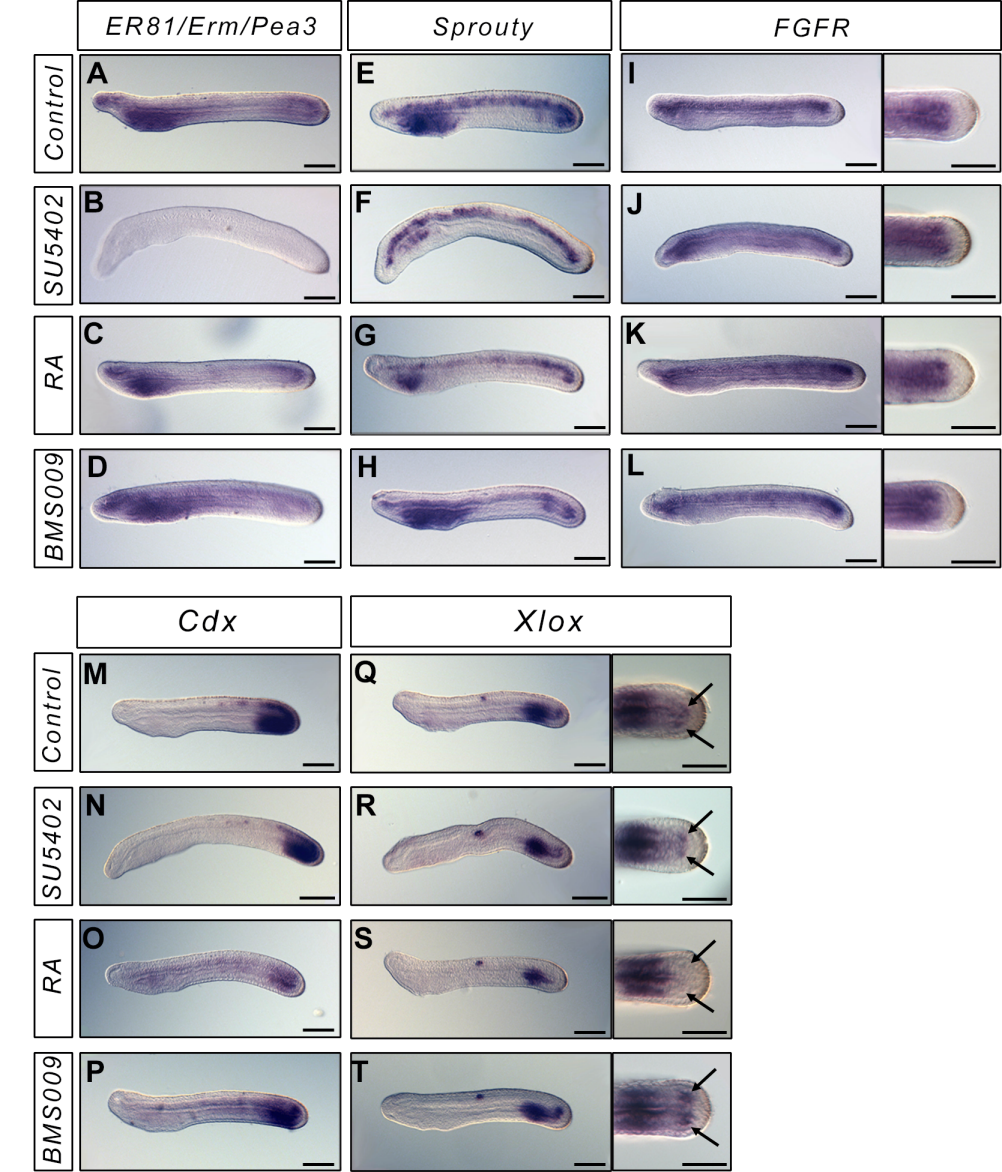
FGF and RA signals do not cross-talk during posterior elongation. Expression of the FGF signalling pathway genes *ER81/Erm/Pea3*, *Sprouty*, *FGFR*, and of the ParaHox genes *Cdx* and *Xlox* in L1 control embryos (A, E, I, M, Q), and in embryos treated at the N3 stage with SU5402 (B, F, J, N, R), RA (C, G, K, O, S) or BMS (D, H, L, P, T) (lateral views, anterior to the left). For *FGFR* and *Xlox*, dorsal views of the tailbud region are also presented. Scale bars = 50μm.

In vertebrates, the position of the "wavefront" in the "clock and wavefront" model for somite formation is controlled by an opposition between RA signalling coming from the most posterior somites, and FGF signalling coming from the tailbud. To test whether we could observe any cross-talk between these pathways in amphioxus during posterior elongation, we looked at the expression of FGF and RA target genes when we interfere with these pathways at the N3 stage. We looked at the expression of *ER81/Erm/Pea3*, *Sprouty*, and *FGFR* as targets of the FGF pathway. After SU5402 treatment, we observe a complete loss of *ER81/Erm/Pea3* expression (Fig 3A and 3B) and a partial loss of *Sprouty* expression (Fig 3E and 3F). Indeed, *Sprouty* is still expressed in the notochord whereas the endodermal expression is lost after treatment (Fig 3E and 3F). On the other hand, following FGFR inhibition, *FGFR* expression is similar to what is observed in control animals (Fig 3I and 3J). When embryos are treated with RA or BMS009, the expression of all these genes is similar to what is observed in wild-type embryos, except in the pharyngeal region which is enlarged in BMS009 treated embryos and reduced in RA treated neurulae (Fig 3C, 3D, 3G, 3H, 3K and 3L). We then looked at the expression of two targets of the RA signalling pathway, the *ParaHox* genes *Cdx* and *Xlox* [27]. We show that in SU5402 treated embryos the expression of both genes is comparable to what is observed in control embryos (Fig 3M, 3N, 3Q and 3R). On the other hand, *Cdx* expression is reduced in RA treated animals (Fig 3M and 3O), and the expression of *Xlox* in the posterior somites is lost (Fig 3Q and 3S). Altogether our data suggest that modifying the RA signalling pathway does not induce modifications in the FGF signal and *vice versa*.

### Correlation between the asymmetric expression of *Nodal* and the asymmetry of somites

**Fig 4:**
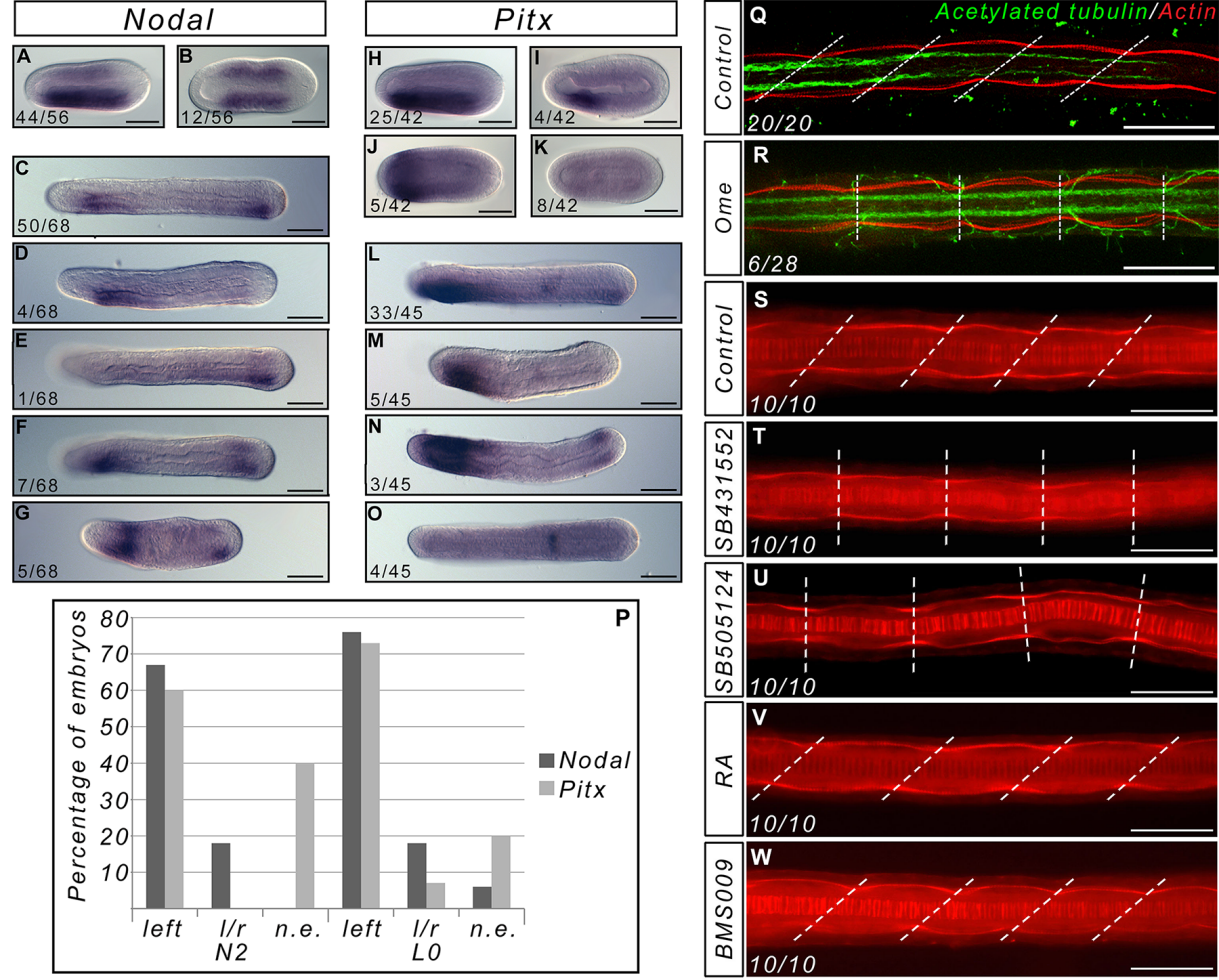
The asymmetry of somites depends on asymmetric *Nodal* expression and is not altered by RA signal pertubations. Expression of *Nodal* in Omeprazole treated embryos at the N2 (A, B) and L0 (C-G) stages (dorsal views, anterior to the left). In wild type embryos expression of *Nodal* is similar to (A) and (C), and expression of *Pitx* is similar to (H) and (L). Expression of *Pitx* in Omeprazole treated embryos at the N2 (H-K) and L0 (L-O) stages (dorsal views, anterior to the left). The number of embryos showing these patterns of expression is indicated on each panel. (P) Graph presenting the number of embryos at the N2 and L0 stage presenting expression in the posterior mesoderm of *Nodal* or *Pitx* only on the left side, on both sides, or on neither side (l/r = left/right, n.e. = not expressed) (P). (Q-R) Texas Red X-Phalloidin (red) and anti-acetylated tubulin (green) labelled control L3 larvae (Q) and Omeprazole treated larvae showing symmetric somites (R). The number of larvae showing symmetric somites after Omeprazole treatment is indicated. Texas Red X-Phalloidin labelling in control (S), SB431552-treated (T), SB505124-treated (U), RA-treated (V) and BMS-treated (W) L3 stage larvae. The pictures represent dorsal views, with anterior to the left, focused on the central region of the larva. The dotted lines join the boundaries between somites on the right and left sides. Scale bars = 50μm.

Somites in amphioxus are asymmetric, with the left somites positioned anteriorly with respect to the right ones. Although the first three somite pairs seem to form simultaneously and appear to be symmetric, the subsequent somites form sequentially with the left somites forming before the right ones. The asymmetric formation of somites starts to be observed when the *Nodal* gene becomes asymmetrically expressed in the mesendoderm [21, 28]. Inhibiting the Nodal signalling pathway induces the formation of symmetric somites in *Branchiostoma floridae* [29]. To test whether or not modifying the asymmetric expression of *Nodal* would have the same effect in *Branchiostoma lanceolatum*, we used Omeprazole, a specific inhibitor of the H+/K+-ATPase [30]. Indeed, it has been shown in vertebrates [31] as in the tunicate *Ciona intestinalis* [32] that inhibiting this ion pump can randomize the asymmetric expression of *Nodal* signalling pathway genes. We tested several time windows and concentrations and checked for the asymmetric expression of *Nodal* and its putative effector *Pitx*. We chose to use a treatment between fertilization until N1 (19 h.p.f. at 19°C), when the blastopore is closed, with 200μM of Omeprazole. Indeed, at lower concentrations we cannot see any effect and continuous treatment at this concentration is lethal. We analyzed the expression of *Nodal* and *Pitx* at different time points from the end of the treatment to the larval stage. At all stages, we observe embryos with posterior expression of these genes on the left side (Fig 4A, 4C, 4E, 4H, 4L and 4P), similar to wild-type embryos, expression on both sides (Fig 4B, 4F, 4G, 4N and 4P) or, in a few cases, no expression (Fig 4D, 4I, 4K, 4M, 4O and 4P). We never observed embryos with expression of *Nodal* or *Pitx* on the right side only. Moreover, the expression of both genes is not always similar for the posterior and the pharyngeal domains: we find some embryos that express *Nodal* on both sides in the tailbud, but only on the left side in the pharynx (Fig 4F), or embryos expressing *Pitx* in the left side of the pharynx but showing no expression in the tailbud (Fig 4M). Overall, almost 20% of the treated embryos show a symmetric expression of *Nodal* in the tailbud (Fig 4P). We also analysed the morphology of the treated embryos at the larval stage and observed that, as for the inhibition of *Nodal* signalling, some larvae (n = 6/28, 21,4%) have symmetric somites (Fig 4Q and 4R). Although we saw a few larvae with two mouths (data not shown), we never observed any larva with the mouth on the right side, nor did we observe larvae with the right somites positioned anteriorly compared to the left ones. Altogether, these data show that inhibition of H+/K+-ATPase activity between fertilization and the end of gastrulation affects the asymmetric expression of both *Nodal* and *Pitx*, which correlates with a modification of the asymmetry of the somites.

### The RA signalling pathway cannot buffer the left/right asymmetry machinery

In vertebrates, RA is able to counteract the influence of the left/right asymmetry machinery [33, 34]. To test whether perturbing the RA pathway would have any influence on somite asymmetry, we compared the morphology of larvae after Nodal pathway inhibition, and after RA and BMS009 treatments. As previously described, when we treated amphioxus embryos at the end of gastrulation with two inhibitors of the type I ALK receptors, SB505124 and SB431552, we obtained larvae with symmetric somites (Fig 4T and 4U) and pharynges [29]. On the other hand, we show that following RA or BMS009 treatments, even when starting treatments as early as the blastula stage, somites are still asymmetric in the larvae, similarly to the control animals (Fig 4V and 4W). Interestingly, the pharynx of RA treated embryos is absent as previously described, showing that the asymmetry of the somites is not the result of mechanical constraints imposed by pharynx morphology.

## Discussion

### A/P patterning and FGF dependency of somite formation

*Hox* genes are known to regulate patterning of embryonic structures along the antero-posterior axis in bilaterians [35]. Moreover, the most anterior limit of *Hox* gene expression in amphioxus, which is the anterior limit of *Hox1* expression [22, 24], corresponds to the limit between somites whose formation is dependent upon FGF signalling and those whose formation does not depend upon this signal [13]. To test whether *Hox1* plays a role in defining a functional boundary between the most anterior and the posterior somites, we shifted its expression anteriorly or posteriorly by interfering with the RA signalling pathway. We show that moving the anterior limit of *Hox1* expression has no effect on the size of the territory in which somites are absent when the FGF signal is inhibited. This means that even if the Hox code might be controlling the antero-posterior identity of the somites in amphioxus, it is not defining their dependency upon an FGF signal. Moreover our data suggest that Hox/RA and FGF signals have independent functions during early embryogenesis in amphioxus.

### The role of RA, FGF, and their negative cross regulation in the control of somitogenesis

In the present view of the "clock and wavefront" model for somitogenesis [3], the boundary between the newly formed somites and the presomitic mesoderm (PSM) is defined by the posterior FGF signal which is antogonized by the RA signal coming from the formed somites and anterior PSM [7, 36]. The FGF signal in the tailbud is thought to be necessary to maintain a pool of undifferentiated cells, whereas RA is promoting the differentiation of PSM cells. FGF and RA signals show opposite gradients and are mutually inhibiting each other. Indeed, FGF8 activates the expression of *Cyp26*, which codes for the enzyme degrading RA, and inhibits the expression of *Raldh2*, the gene coding for the enzyme responsible for the synthesis of RA [7]. On the other hand, RA can restrict the expression of *fgf8* in chicken [7] and mouse [37, 38], or activates the expression of *MKP3*, which codes for a phosphatase that blocks the MAPK cascade activation in *Xenopus* [39]. This negative crosstalk between FGF and RA is not only important during somitogenesis, but has been recruited in many developmental processes such as the antero-posterior patterning of the heart field [40], the timing of emigration of trunk neural crest cells [41], or limb induction [42]. We show that, in amphioxus, the RA signalling pathway is not implicated in somitogenesis, neither through interaction with the FGF signal for anterior somite formation, nor during posterior elongation. Indeed, activating or inhibiting the RA pathway at early stages or during posterior elongation leads to normal number and shape of formed somites. This is a major difference with what is observed in all vertebrates studied thus far. Indeed, RA depletion induces the formation of smaller somites in quail [43], chicken [7], mouse [44], and *Xenopus* [39], whereas RA signalling activation induces caudal truncation in mouse [45] and zebrafish [46], as well as abnormal somite size and disorganized somite boundaries in *Xenopus* [39].

In this work we also show that the cross regulation between the FGF and RA signalling pathways is absent in amphioxus. This raises questions about how and when the involvement of both pathways in somitogenesis evolved in the chordate clade, as well as of crosstalk between them. In tunicates, the sister group of vertebrates, the role of RA during development seems to be different in the different lineages. Ascidians like *Ciona intestinalis* possess a unique retinoic acid receptor (RAR); upon activation of the RA signalling pathway, *Hox1* expression shifts anteriorly in the epidermis and nervous system [47, 48] suggesting a conserved role for RA in controlling the expression of Hox genes in chordates. However, the larvacean *Oikopleura dioica* possesses no RAR [49] and no homeotic transformation is observed when embryos are treated with RA, suggesting that the formation of the chordate body plan can be achieved without the contribution of a classical RA signalling pathway [49]. In any case, although tunicate tadpoles have segmented muscles, these are not formed through a process comparable to somitogenesis [10]. Moreover, there is no evidence for a putative role of RA in the formation of these cells, although *Raldh2*, the gene coding for the enzyme responsible for the synthesis of RA, is expressed in the most anterior muscle cells in *Ciona* [47]. As for the FGF signal, it has been shown that it is necessary for heart specification at early stages [50]. It has also been shown that FGF9/16/20 blocks the expression of *Ci-MRF*, and thus the myogenesis process, in the mesenchyme cells [51], suggesting that FGF rather functions as a pro-cardiac and anti-muscle signal in urochordates. Concerning the interaction between RA and FGF signalling pathways, it has been shown that in *Ciona* they negatively interact to pattern the posterior epidermis [52], suggesting that RA/FGF negative crosstalk might have appeared in the ancestor of tunicates and vertebrates. From our data and what we know in tunicates, we propose that even if it is most likely that the RA/FGF cross regulation appeared in the ancestor of olfactores (tunicates plus vertebrates), their respective role and their interaction in the control of somitogenesis are vertebrate novelties.

### Somitogenesis and left/right asymmetry

Vertebrates are externally symmetrical but the position of visceral and abdominal organs derived from the endoderm is completely asymmetric. Morphological asymmetry is common in bilaterians, and Nodal signalling is a key actor in controlling laterality [53]. It has recently been shown that a *Nodal-related* gene is also defining laterality in a cnidarian [54] suggesting that the control of axial asymmetry along the main body axes (antero-posterior or oral-aboral) by the Nodal pathway might be ancestral in eumetazoans. The apparent symmetry of vertebrates is mainly the result of the symmetrical formation of somites, which ensures the symmetric morphogenesis of axial skeleton and skeletal muscles. In 2005, three research groups showed that bilaterally symmetric somites is not the default state in vertebrates, and that RA is responsible for the buffering of the lateralizing machinery in the PSM [33, 34, 37]. The main role of RA seems to be, in the case of vertebrates, to synchronize the oscillation of clock gene expression between the left and right sides of the embryo, although through different strategies [33, 34, 37]. In amphioxus, somites are asymmetric, with the left somites more anterior than the right ones. It has recently been shown that inhibiting the Nodal signalling pathway after gastrulation, when *Nodal*, *Pitx* or *Cerberus* expression start to be asymmetric [21, 28, 55–57], leads to the symmetric formation of somites, suggesting that somitogenesis is affected by a conserved Nodal pathway laterality signal [29]. In this study we also show that the modification of *Nodal* and *Pitx* asymmetric expression through the inhibition of the H+/K+-ATPase correlates with the alteration of asymmetric somite formation. In cephalochordates, the RA signal is not active in the posterior part of the body where somites form during posterior elongation [25]. One hypothesis to explain the differences observed with vertebrates is that in amphioxus the distance between the region where the RA signal is active and the territory in which somitogenesis occurs is too large to render RA able to buffer the left/right asymmetry cue. However, treating amphioxus embryos with RA moves the expression of the unique RAR posteriorly, making the tailbud sensitive to RA applied to the embryos [25]. We observe that even if we interfere with the RA pathway, somites still form in an asymmetric manner in amphioxus, demonstrating that the RA signal cannot buffer the lateralizing influence of the left/right machinery.

### Hypothesis for the evolution of somitogenesis in chordates

If we compare the somitogenesis process between cephalochordates and vertebrates, several morphological differences should be pointed out. First, as previously mentioned, the mesoderm of the vertebrate head is not segmented, whereas amphioxus somites extend the whole length of the embryo. Second, in amphioxus the somites do not form through a mesenchyme-epithelium transition, but directly from epithelial structures (the archenteron roof for enterocoelic somites, and the tailbud for the schizocoelic somites). Moreover, during formation of the posterior somites, there is no intermediate tissue between the tailbud and the last formed somites in cephalochordates (i.e. there is no PSM). Finally, somites are asymmetric in amphioxus whereas they form symmetrically in vertebrates.

**Fig 5:**
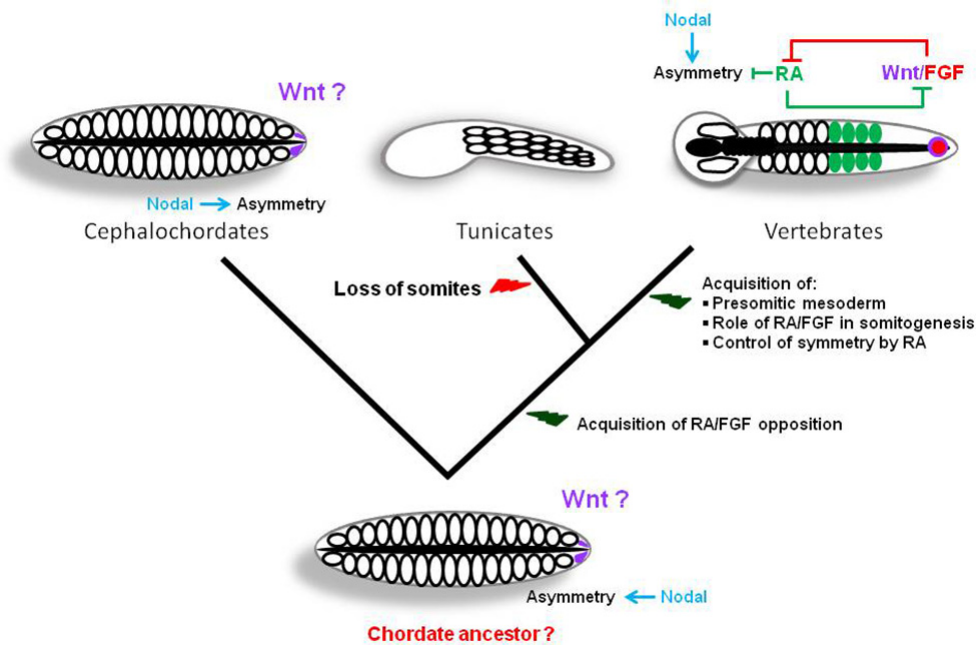
Evolutionary scenario for somitogenesis in chordates. Evolutionary relationships among the three chordate clades are presented, as well as a schematic view of the morphology of embryos of the putative ancestor of chordates, cephalochordates, tunicates and vertebrates (all dorsal views except tunicates for which a lateral view is schematized) during posterior elongation. We propose that the ancestral chordate embryo was morphologically close to amphioxus and that the asymmetry of somite formation was under the control of Nodal. After the divergence of cephalochordates, an opposition between the RA and FGF pathways was acquired. In tunicates, the somitogenesis process was lost, probably as an adaptation to a reduced number of embryonic cells. In the vertebrate lineage, the opposition between RA and FGF gained importance in parallel to the acquisition of the PSM as an intermediate zone between both signals. The recruitment of RA in the control of somitogenesis permitted the acquisition of symmetry through the buffering of the left/right machinery controlled by Nodal. The Wnt pathway, indicated with a question mark, is a good candidate as a signal ancestrally controlling posterior elongation in chordates although up to now there are no functional data supporting this hypothesis.

Interestingly, the PSM defines a territory where RA and FGF gradients are negatively regulating each other, and we show in this study that such opposition is absent in amphioxus. With respect to the role of RA, we show that, in contrast to vertebrates, this signal is not implicated at all during the somitogenesis process in amphioxus: it does not interact with FGF, and it is not able to buffer the lateralizing signal of the left/right machinery. Considering the fact that a negative cross-regulation between RA and FGF was described in the tunicate *Ciona intestinalis* [52], and hypothesising that the control of somitogenesis in the ancestor of chordates was likely similar to that in amphioxus, we can propose a scenario for the evolution of somitogenesis in chordates (Fig 5). The ancestor of olfactores acquired a functional cross-talk between RA and FGF that gained greater significance during embryonic development in the vertebrate lineage, probably due to the acquisition of three RARs *versus* one, and of four FGFRs and twenty-two ligands *versus* a unique FGFR and seven to eight ligands in non-vertebrate chordates [58]. In vertebrates, the RA signal was recruited in the control of somitogenesis together with FGF in parallel to the formation of an intermediate structure between both signals, the PSM. Moreover, the implication of RA in somitogenesis also permitted the acquisition of the symmetrical character of this process through the buffering of the left/right signal. The main signal controlling posterior elongation in the chordate ancestor is however still unknown. The expression of several genes coding for actors of the Wnt signalling pathway in the tailbud during amphioxus posterior elongation [11, 12, 59–62], together with data in vertebrates, make this signal a good candidate.

Several lines of evidence support the hypothesis that the ancestor of chordates had amphioxus-like somitogenesis. From paleontological data it has been suggested that the ancestor of chordates was segmented from the most anterior to the most posterior part of the body like amphioxus [63]. Although there is up to now no data suggesting that these segments were asymmetric, the fact that in amphioxus the asymmetry of somitogenesis is controlled by the Nodal signalling pathway and that this signal is also controlling left/right asymmetries in other bilaterians [53], combined with evidence that the default state in vertebrates is asymmetry [33, 34, 37] support this proposition. Our scenario also implies that at least part of the "clock and wavefront" system for the control of somite formation was acquired in the vertebrate lineage. Recent experiments in quail have shown that non-somitic mesoderm is able to form synchronously several somites without any external cues except deprivation of BMP signal [64]. The authors suggest that the "clock and wavefront" system only serves to control the timing of somite formation and their rostro-caudal patterning. In amphioxus, we show here that there is no wavefront comparable to the one of vertebrates. Indeed, modification of FGF signalling does not alter posterior somitogenesis. Moreover, the RA pathway is not acting as an opposing signal to the FGF pathway. Furthermore, even if segmentation genes are expressed during somitogenesis [12] there is up to now no evidence for a clock. If our hypothesis is true, the recruitment of RA and FGF in the control of somitogenesis was key to the evolution of vertebrate morphology. Indeed, it allowed the acquisition of an important plasticity in the formation of the posterior part of the embryo [65] and also probably the appearance of lateral locomotor structures like fins or limbs, since such morphological traits would probably not have been selected during evolution in animals with asymmetric somites.

## References

[1] Dequeant ML, Pourquie O. Segmental patterning of the vertebrate embryonic axis. Nat Rev Genet. 2008;9(5):370–82. 10.1038/nrg2320 18414404

[2] Wilson V, Olivera-Martinez I, Storey KG. Stem cells, signals and vertebrate body axis extension. Development. 2009;136(10):1591–604. 10.1242/dev.021246 19395637

[3] Cooke J, Zeeman EC. A clock and wavefront model for control of the number of repeated structures during animal morphogenesis. J Theor Biol. 1976;58(2):455–76. . 940335 10.1016/s0022-5193(76)80131-2

[4] Aulehla A, Herrmann BG. Segmentation in vertebrates: clock and gradient finally joined. Genes Dev. 2004;18(17):2060–7. . 15342488 10.1101/gad.1217404

[5] Hubaud A, Pourquie O. Signalling dynamics in vertebrate segmentation. Nat Rev Mol Cell Biol. 2014;15(11):709–21. 10.1038/nrm3891 25335437

[6] Pourquie O. Vertebrate segmentation: from cyclic gene networks to scoliosis. Cell. 2011;145(5):650–63. 10.1016/j.cell.2011.05.011 21620133 PMC3164975

[7] Diez del Corral R, Olivera-Martinez I, Goriely A, Gale E, Maden M, Storey K. Opposing FGF and retinoid pathways control ventral neural pattern, neuronal differentiation, and segmentation during body axis extension. Neuron. 2003;40(1):65–79. . 14527434 10.1016/s0896-6273(03)00565-8

[8] Delsuc F, Brinkmann H, Chourrout D, Philippe H. Tunicates and not cephalochordates are the closest living relatives of vertebrates. Nature. 2006;439(7079):965–8. . 16495997 10.1038/nature04336

[9] Satoh N, Rokhsar D, Nishikawa T. Chordate evolution and the three-phylum system. Proc Biol Sci. 2014;281(1794):20141729 10.1098/rspb.2014.1729 25232138 PMC4211455

[10] Passamaneck YJ, Hadjantonakis AK, Di Gregorio A. Dynamic and polarized muscle cell behaviors accompany tail morphogenesis in the ascidian Ciona intestinalis. PLoS One. 2007;2(8):e714 . 17684560 10.1371/journal.pone.0000714 PMC1934933

[11] Schubert M, Holland LZ, Stokes MD, Holland ND. Three amphioxus Wnt genes (AmphiWnt3, AmphiWnt5, and AmphiWnt6) associated with the tail bud: the evolution of somitogenesis in chordates. Dev Biol. 2001;240(1):262–73. . 11784062 10.1006/dbio.2001.0460

[12] Beaster-Jones L, Kaltenbach SL, Koop D, Yuan S, Chastain R, Holland LZ. Expression of somite segmentation genes in amphioxus: a clock without a wavefront? Dev Genes Evol. 2008;218(11–12):599–611. 10.1007/s00427-008-0257-5 18949486

[13] Bertrand S, Camasses A, Somorjai I, Belgacem MR, Chabrol O, Escande ML, et al Amphioxus FGF signaling predicts the acquisition of vertebrate morphological traits. Proc Natl Acad Sci U S A. 2011;108(22):9160–5. 10.1073/pnas.1014235108 21571634 PMC3107284

[14] Schubert M, Yu JK, Holland ND, Escriva H, Laudet V, Holland LZ. Retinoic acid signaling acts via Hox1 to establish the posterior limit of the pharynx in the chordate amphioxus. Development. 2005;132(1):61–73. . 15576409 10.1242/dev.01554

[15] Schubert M, Holland ND, Laudet V, Holland LZ. A retinoic acid-Hox hierarchy controls both anterior/posterior patterning and neuronal specification in the developing central nervous system of the cephalochordate amphioxus. Dev Biol. 2006;296(1):190–202. . 16750825 10.1016/j.ydbio.2006.04.457

[16] Fuentes M, Benito E, Bertrand S, Paris M, Mignardot A, Godoy L, et al Insights into spawning behavior and development of the European amphioxus (Branchiostoma lanceolatum). J Exp Zool B Mol Dev Evol. 2007;308(4):484–93. . 17520703 10.1002/jez.b.21179

[17] Fuentes M, Schubert M, Dalfo D, Candiani S, Benito E, Gardenyes J, et al Preliminary observations on the spawning conditions of the European amphioxus (Branchiostoma lanceolatum) in captivity. J Exp Zool B Mol Dev Evol. 2004;302(4):384–91. . 15287102 10.1002/jez.b.20025

[18] Hirakow R, Kajita N. Electron microscopic study of the development of amphioxus, Branchiostoma belcheri tsingtauense: The gastrula. Journal of Morphology. 1991;207(1):37–52. 29865496 10.1002/jmor.1052070106

[19] Hirakow R, Kajita N. Electron microscopic study of the development of amphioxus, Branchiostoma belcheri tsingtauense: the neurula and larva. Kaibogaku Zasshi. 1994;69(1):1–13. . 8178614

[20] Holland LZ, Holland PWH, Holland ND. Revealing homologies between body parts of distantly related animals by in situ hybridization to developmental genes: Amphioxus vs. vertebrates In: Ferraris JD, Palumbi S, editors. *Molecular Zoology*: *Advances*, *Strategies*, *and Protocols*. New York: Wiley-Liss; 1996.

[21] Somorjai I, Bertrand S, Camasses A, Haguenauer A, Escriva H. Evidence for stasis and not genetic piracy in developmental expression patterns of Branchiostoma lanceolatum and Branchiostoma floridae, two amphioxus species that have evolved independently over the course of 200 Myr. Dev Genes Evol. 2008;218(11–12):703–13. 10.1007/s00427-008-0256-6 18843503

[22] Pascual-Anaya J, Adachi N, Alvarez S, Kuratani S, D'Aniello S, Garcia-Fernandez J. Broken colinearity of the amphioxus Hox cluster. Evodevo. 2012;3(1):28 10.1186/2041-9139-3-28 23198682 PMC3534614

[23] Wu HR, Chen YT, Su YH, Luo YJ, Holland LZ, Yu JK. Asymmetric localization of germline markers Vasa and Nanos during early development in the amphioxus Branchiostoma floridae. Dev Biol. 2011;353(1):147–59. 10.1016/j.ydbio.2011.02.014 21354126

[24] Holland PW, Holland LZ, Williams NA, Holland ND. An amphioxus homeobox gene: sequence conservation, spatial expression during development and insights into vertebrate evolution. Development. 1992;116(3):653–61. . 1363226 10.1242/dev.116.3.653

[25] Escriva H, Holland ND, Gronemeyer H, Laudet V, Holland LZ. The retinoic acid signaling pathway regulates anterior/posterior patterning in the nerve cord and pharynx of amphioxus, a chordate lacking neural crest. Development. 2002;129(12):2905–16. . 12050138 10.1242/dev.129.12.2905

[26] Mohammadi M, McMahon G, Sun L, Tang C, Hirth P, Yeh BK, et al Structures of the tyrosine kinase domain of fibroblast growth factor receptor in complex with inhibitors. Science. 1997;276(5314):955–60. . 9139660 10.1126/science.276.5314.955

[27] Osborne PW, Benoit G, Laudet V, Schubert M, Ferrier DE. Differential regulation of ParaHox genes by retinoic acid in the invertebrate chordate amphioxus (Branchiostoma floridae). Dev Biol. 2009;327(1):252–62. 10.1016/j.ydbio.2008.11.027 19103191

[28] Yu JK, Holland LZ, Holland ND. An amphioxus nodal gene (AmphiNodal) with early symmetrical expression in the organizer and mesoderm and later asymmetrical expression associated with left-right axis formation. Evol Dev. 2002;4(6):418–25. . 12492142 10.1046/j.1525-142x.2002.02030.x

[29] Soukup V, Yong LW, Lu TM, Huang SW, Kozmik Z, Yu JK. The Nodal signaling pathway controls left-right asymmetric development in amphioxus. Evodevo. 2015;6:5 10.1186/2041-9139-6-5 25954501 PMC4423147

[30] Morii M, Takeguchi N. Different biochemical modes of action of two irreversible H+,K(+)-ATPase inhibitors, omeprazole and E3810. J Biol Chem. 1993;268(29):21553–9. . 8408006

[31] Levin M, Thorlin T, Robinson KR, Nogi T, Mercola M. Asymmetries in H+/K+-ATPase and cell membrane potentials comprise a very early step in left-right patterning. Cell. 2002;111(1):77–89. . 12372302 10.1016/s0092-8674(02)00939-x

[32] Shimeld SM, Levin M. Evidence for the regulation of left-right asymmetry in Ciona intestinalis by ion flux. Dev Dyn. 2006;235(6):1543–53. . 16586445 10.1002/dvdy.20792

[33] Vermot J, Pourquie O. Retinoic acid coordinates somitogenesis and left-right patterning in vertebrate embryos. Nature. 2005;435(7039):215–20. . 15889094 10.1038/nature03488

[34] Kawakami Y, Raya A, Raya RM, Rodriguez-Esteban C, Izpisua Belmonte JC. Retinoic acid signalling links left-right asymmetric patterning and bilaterally symmetric somitogenesis in the zebrafish embryo. Nature. 2005;435(7039):165–71. . 15889082 10.1038/nature03512

[35] Pearson JC, Lemons D, McGinnis W. Modulating Hox gene functions during animal body patterning. Nat Rev Genet. 2005;6(12):893–904. . 16341070 10.1038/nrg1726

[36] Dubrulle J, McGrew MJ, Pourquie O. FGF signaling controls somite boundary position and regulates segmentation clock control of spatiotemporal Hox gene activation. Cell. 2001;106(2):219–32. . 11511349 10.1016/s0092-8674(01)00437-8

[37] Vermot J, Gallego Llamas J, Fraulob V, Niederreither K, Chambon P, Dolle P. Retinoic acid controls the bilateral symmetry of somite formation in the mouse embryo. Science. 2005;308(5721):563–6. . 15731404 10.1126/science.1108363

[38] Kumar S, Duester G. Retinoic acid controls body axis extension by directly repressing Fgf8 transcription. Development. 2014;141(15):2972–7. 10.1242/dev.112367 25053430 PMC4197666

[39] Moreno TA, Kintner C. Regulation of segmental patterning by retinoic acid signaling during Xenopus somitogenesis. Dev Cell. 2004;6(2):205–18. . 14960275 10.1016/s1534-5807(04)00026-7

[40] Sirbu IO, Zhao X, Duester G. Retinoic acid controls heart anteroposterior patterning by down-regulating Isl1 through the Fgf8 pathway. Dev Dyn. 2008;237(6):1627–35. 10.1002/dvdy.21570 18498088 PMC2614402

[41] Martinez-Morales PL, Diez del Corral R, Olivera-Martinez I, Quiroga AC, Das RM, Barbas JA, et al FGF and retinoic acid activity gradients control the timing of neural crest cell emigration in the trunk. J Cell Biol. 2011;194(3):489–503. 10.1083/jcb.201011077 21807879 PMC3153641

[42] Zhao X, Sirbu IO, Mic FA, Molotkova N, Molotkov A, Kumar S, et al Retinoic acid promotes limb induction through effects on body axis extension but is unnecessary for limb patterning. Curr Biol. 2009;19(12):1050–7. 10.1016/j.cub.2009.04.059 19464179 PMC2701469

[43] Maden M, Graham A, Zile M, Gale E. Abnormalities of somite development in the absence of retinoic acid. Int J Dev Biol. 2000;44(1):151–9. . 10761860

[44] Niederreither K, Subbarayan V, Dolle P, Chambon P. Embryonic retinoic acid synthesis is essential for early mouse post-implantation development. Nat Genet. 1999;21(4):444–8. . 10192400 10.1038/7788

[45] Sakai Y, Meno C, Fujii H, Nishino J, Shiratori H, Saijoh Y, et al The retinoic acid-inactivating enzyme CYP26 is essential for establishing an uneven distribution of retinoic acid along the anterio-posterior axis within the mouse embryo. Genes Dev. 2001;15(2):213–25. . 11157777 10.1101/gad.851501 PMC312617

[46] Martin BL, Kimelman D. Brachyury establishes the embryonic mesodermal progenitor niche. Genes Dev. 2010;24(24):2778–83. 10.1101/gad.1962910 21159819 PMC3003196

[47] Nagatomo K, Fujiwara S. Expression of Raldh2, Cyp26 and Hox-1 in normal and retinoic acid-treated Ciona intestinalis embryos. Gene Expr Patterns. 2003;3(3):273–7. . 12799071 10.1016/s1567-133x(03)00051-6

[48] Nagatomo K, Ishibashi T, Satou Y, Satoh N, Fujiwara S. Retinoic acid affects gene expression and morphogenesis without upregulating the retinoic acid receptor in the ascidian Ciona intestinalis. Mech Dev. 2003;120(3):363–72. . 12591605 10.1016/s0925-4773(02)00441-0

[49] Canestro C, Postlethwait JH. Development of a chordate anterior-posterior axis without classical retinoic acid signaling. Dev Biol. 2007;305(2):522–38. . 17397819 10.1016/j.ydbio.2007.02.032

[50] Davidson B, Shi W, Beh J, Christiaen L, Levine M. FGF signaling delineates the cardiac progenitor field in the simple chordate, Ciona intestinalis. Genes Dev. 2006;20(19):2728–38. . 17015434 10.1101/gad.1467706 PMC1578698

[51] Imai KS, Levine M, Satoh N, Satou Y. Regulatory blueprint for a chordate embryo. Science. 2006;312(5777):1183–7. . 16728634 10.1126/science.1123404

[52] Pasini A, Manenti R, Rothbacher U, Lemaire P. Antagonizing retinoic acid and FGF/MAPK pathways control posterior body patterning in the invertebrate chordate Ciona intestinalis. PLoS One. 2013;7(9):e46193 . 23049976 10.1371/journal.pone.0046193 PMC3458022

[53] Namigai EK, Kenny NJ, Shimeld SM. Right across the tree of life: the evolution of left-right asymmetry in the Bilateria. Genesis. 2014;52(6):458–70. 10.1002/dvg.22748 24510729

[54] Watanabe H, Schmidt HA, Kuhn A, Hoger SK, Kocagoz Y, Laumann-Lipp N, et al Nodal signalling determines biradial asymmetry in Hydra. Nature. 2014;515(7525):112–5. 10.1038/nature13666 25156256

[55] Boorman CJ, Shimeld SM. Pitx homeobox genes in Ciona and amphioxus show left-right asymmetry is a conserved chordate character and define the ascidian adenohypophysis. Evol Dev. 2002;4(5):354–65. . 12356265 10.1046/j.1525-142x.2002.02021.x

[56] Le Petillon Y, Oulion S, Escande ML, Escriva H, Bertrand S. Identification and expression analysis of BMP signaling inhibitors genes of the DAN family in amphioxus. Gene Expr Patterns. 2013;13(8):377–83. 10.1016/j.gep.2013.07.005 23872339

[57] Onai T, Yu JK, Blitz IL, Cho KW, Holland LZ. Opposing Nodal/Vg1 and BMP signals mediate axial patterning in embryos of the basal chordate amphioxus. Dev Biol. 2010;344(1):377–89. 10.1016/j.ydbio.2010.05.016 20488174 PMC4781670

[58] Oulion S, Bertrand S, Escriva H. Evolution of the FGF Gene Family. Int J Evol Biol. 2012;2012:298147 10.1155/2012/298147 22919541 PMC3420111

[59] Lin HC, Holland LZ, Holland ND. Expression of the AmphiTcf gene in amphioxus: insights into the evolution of the TCF/LEF gene family during vertebrate evolution. Dev Dyn. 2006;235(12):3396–403. . 17013891 10.1002/dvdy.20971

[60] Qian G, Li G, Chen X, Wang Y. Characterization and embryonic expression of four amphioxus Frizzled genes with important functions during early embryogenesis. Gene Expr Patterns. 2013;13(8):445–53. 10.1016/j.gep.2013.08.003 24012522

[61] Holland LZ, Holland NN, Schubert M. Developmental expression of AmphiWnt1, an amphioxus gene in the Wnt1/wingless subfamily. Dev Genes Evol. 2000;210(10):522–4. . 11180802 10.1007/s004270000089

[62] Schubert M, Holland LZ, Holland ND. Characterization of an amphioxus wnt gene, AmphiWnt11, with possible roles in myogenesis and tail outgrowth. Genesis. 2000;27(1):1–5. . 10862149 10.1002/1526-968x(200005)27:1<1::aid-gene10>3.0.co;2-3

[63] Morris SC, Caron JB. Pikaia gracilens Walcott, a stem-group chordate from the Middle Cambrian of British Columbia. Biol Rev Camb Philos Soc. 2012;87(2):480–512. 10.1111/j.1469-185X.2012.00220.x 22385518

[64] Dias AS, de Almeida I, Belmonte JM, Glazier JA, Stern CD. Somites without a clock. Science. 2014;343(6172):791–5. 10.1126/science.1247575 24407478 PMC3992919

[65] Keyte AL, Smith KK. Heterochrony and developmental timing mechanisms: changing ontogenies in evolution. Semin Cell Dev Biol. 2014;34:99–107. 10.1016/j.semcdb.2014.06.015 24994599 PMC4201350

